# Diversity and Species Composition of Microbiota Associated with Mosquito Breeding Habitats: A Study from Kurunegala District in Sri Lanka

**DOI:** 10.1155/2019/5897317

**Published:** 2019-12-29

**Authors:** L. D. Amarasinghe, H. A. K. Ranasinghe

**Affiliations:** Department of Zoology and Environmental Management, Faculty of Science, University of Kelaniya, Dalugama GQ 11600, Sri Lanka

## Abstract

The pool of microbiota associated with mosquito breeding habitats varies with the habitat type and its characteristic features. The pool of microbiota in a given mosquito breeding habitat can include free living, symbiotic, noncompetitive, parasitic, predatory, and toxin producing species. However, in Sri Lanka the studies on the microbiota associated with mosquito breeding habitats are scarce. The present study was conducted to identify microbiota species/taxa associated with a variety of mosquito breeding habitats in selected areas of the Kurunegala district in Sri Lanka to determine the relationship, if any, the microbiota has with mosquito larvae breeding. A total of 44 microbiota species/taxa belonging to 10 phyla, namely, Bacillariophyta, Charophyta, Chlorophyta, Cyanobacteria/Cyanophyta, Ochrophyta/Heterokontophyta, Amoebozoa, Euglenozoa, Ciliophora, Arthropoda, and Rotifera were identified. *Vorticella microstoma* (Ciliophora) showed a constant occurrence frequency in rice field habitats occupied mainly by *Culex tritaeniorhynchus* while the rest of the species had an accidental or rare frequency of occurrence. Nineteen species/taxa were identified as common species. Trophont stages of *Vorticella microstoma* and *Zoothamnium* spp. were found attached to the cuticle of mosquito larvae but only *V. microstoma* caused a lethal effect. The autotrophic protist, *Euglena geniculate*, *Closterium* spp., and *Pinnularia* spp. served as the diet items to mosquito larvae. The majority of the microbiota identified had no observable effect on mosquito larvae breeding.

## 1. Introduction

Mosquito borne diseases are among the major health problems in almost all tropical and subtropical countries including Sri Lanka [1]. Gunathilaka [2] updated the existing mosquito catalogue prepared by Amerasinghe [3] to include 159 mosquito species under 19 genera in Sri Lanka of which about ten species recorded to serve as main vectors of human diseases. Preference for breeding habitat by adult female mosquitoes for oviposition varies from large permanent water bodies such as boundaries of lake edges, ponds, river banks, marshy lands to small temporary water sources such as water accumulated burrow pits, tree holes, and small containers depending on the species of the mosquito. Mosquito larvae development vary with varying levels of abiotic parameters such as turbidity, level of dissolved organic and inorganic matter, levels of dissolved oxygen, light intensity, amount of shade of the habitat [4], and biotic parameters such as the presence of the potential predators, parasites, or competitors [5–8]. The presence of competitors and predators in a mosquito larval habitat may reduce larval survival due to sharing and competing for the same food source or preying on mosquito larvae, respectively [9, 10]. Marten [11] has reported higher abundance of mosquito larvae in places where phytoplanktons such as diatoms, desmids, and green algae such as *Spirogyra* spp. are common. There are at least 200 species of phytoplanktons associated with mosquito breeding habitats and larval instars extensively feed upon them [12, 13]. However, species such as *Kirchneriella*, *Scenedesmus*, *Coelastrum*, *Selenastrum*, *Dactylococcus,* and *Tetrallantos* were found to be virtually indigestible by mosquito larvae, hence reducing the survival of certain species of mosquito larvae [11, 14]. The presence of predatory, parasitic, and toxic species of microbiota associated with oviposition sites of a variety of mosquito species has been reported previously [13, 15–17, 18]. To date, only a small number of species of the microbiota that inhabit in mosquito breeding habitats have been recorded from Sri Lanka [9, 19]. Studies on microbiota assemblage in relation to diverse vector mosquito breeding habitats and their association with mosquito larvae are scarce. Such studies may help developing strategies for the management of vector mosquito larvae; hence, this study was conducted to study the species diversity and species composition of microbiota association with mosquito breeding habitats.

## 2. Methodology

### 2.1. Study Area

**Figure 1:**
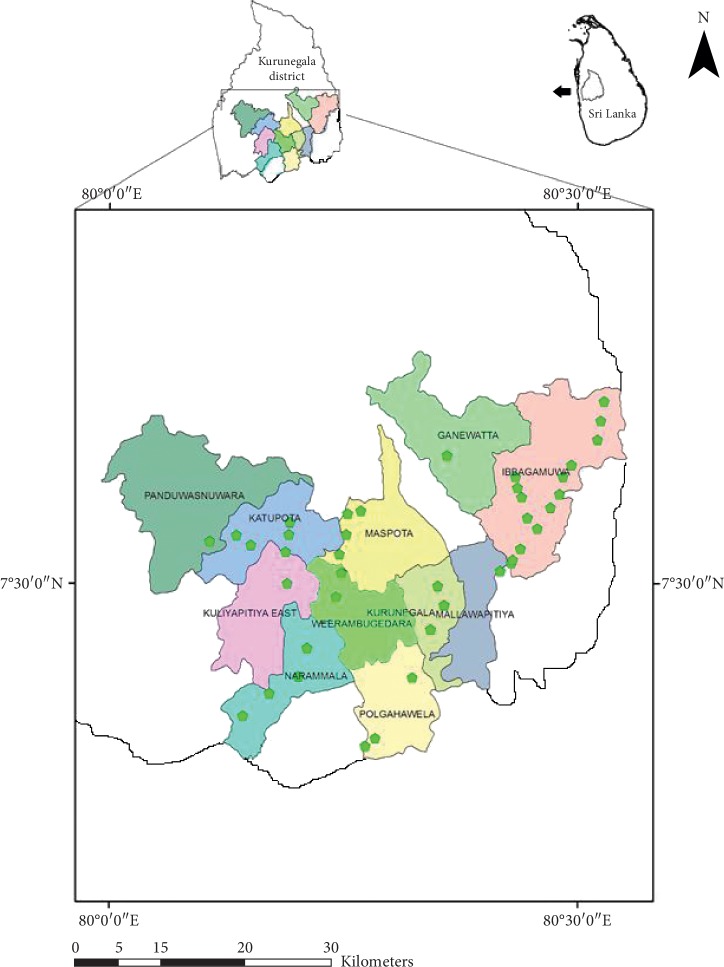
Divisional secretariat divisions (DSD) of the Kurunegala district showing the sampling sites.

This study was performed in Kurunegala district in North Western Province of Sri Lanka. The sampling area included eleven administrative Divisions, that is, Divisional Secretariat Divisions (DSD), namely Ibbagamuwa, Kurunegala, Kuliyapitiya, Polgahawela, Narammala, Panduwasnuwara, Katupotha, Maspota, Ganewatta, Weerambugedara, and Mallawapitiya in 1367.5 km^2^ area (Figure 1). In Kurunegala district the average annual temperature is 27.3°C. During the month of March the temperature rises up to about 34°C. The average annual rainfall is 152.5 mm. A major change in the weather of Kurunegala district occurs during the monsoons from April to June and October to December, the times of the year where heavy rains are expected. The main climatic features of the Kurunegala district is given in Table 1.

### 2.2. Sample Collection and Identification of Mosquito Larvae and Microbiota

Sampling was performed bimonthly from September 2017 to August 2018. Forty mosquito breeding habitats were identified and geo-referenced (GARMIN-etrex SUMMIT). Water samples were collected using dipping, siphoning, and pipetting methods according to the nature of the breeding habitats (National Dengue Control Unit, Sri Lanka). For the dipping method, a metal scooper (250 mL with a 30 cm long handle) was held vertically in the shallow area of a water body and a sample of water was taken maximum at the handle depth to comprise subsurface and bottom layers. When dipping is impossible; in small and flat water sources, sampling was performed by pipetting out the water using a pasture pipette; siphoning was done in places such as tree holes and tyres. The water sample collection from individual habitat was decanted into a larval rearing transparent plastic containers (11.5 cm width, 15 cm height). Then, equal volumes were transferred into three larval rearing transparent containers (6.5 cm width, 12 cm height) through a loosely fitted piece of mosquito net. Live mosquito larvae retained on the net were collected into a container and the lid was screwed loosely during the transportation to the laboratory. This procedure was repeated for all the habitats at every sampling.

## 3. Sample Analysis

### 3.1. Estimation of Microbiota Abundance

Two water containers free of mosquito larvae were fixed *in situ*: one using Rose Bengal solution (5% formalin with 0.04% Rose Bengal stain) to preserve nonprotist eukaryotes and the other using 5% Lugols' to preserve bacterioplankton and phytoplankton. This was repeated at every habitat at every sampling. Sample containers were brought to the laboratory. The number of protists and other eukaryotes (Zooplankton) was estimated in Sedgwick–Rafter chambers (50 mm length, 20 mm width, 1 mm deep) through longitudinal transects under the compound microscope (×100 magnification) (OLYMPUS × C21). HYDRO-BIOS phytoplankton chamber (dimensions; 33 × 33 mm, thickness; 1 mL) was used to estimate the phytoplankton according to the method described by [20, 21]. The units, cells, colonies, and filaments were enumerated until the number of individuals of the dominant species reached a total of at least 100 as described by Lund et al. [22].

The microbiota were identified to taxa/species level using temporary slide mounts on diluted canadabalsm. Identification was done at ×400 magnification using standard identification keys and pictorial guides [23–25].

### 3.2. Survival of Mosquito Larvae and the Effect of Microbiota on Larvae Rearing

The water containers retained with mosquito larvae were carefully brought to the laboratory nonpreserved and in fresh condition. Five to eight numbers of 3^rd^ or 4^th^ instar larvae were carefully siphoned off using a pasture pipette and transferred into a separate glass vial with 70% ethanol. Larvae were identified to species level by morphological features observed under the stereomicroscope [26–28].

Rearing containers with remaining live mosquito larvae collected from individual habitats were maintained in the laboratory at room temperature (27 ± 2°C). Lid of the containers were replaced with a small-sized mosquito net for live observations. Observations on larval activity and development were continued for up to ten days or until pupation.

### 3.3. Data Analysis

Dynamics of mosquito larvae population encountered in the sampling sites were expressed according to the formula, *C*°=°(*n*/*N*)°∗°100 where *C* is distribution, *n is the* number of sites of the species, and *N* is the number of all sites. The distribution classes accepted by [29], C1: sporadic appearance (constancy 0–20%); C2: infrequent (20.1–40%); C3: moderate (40.1–60%); C4: frequent (60.1–80%); C5: constant (80.1–100%), were adopted.

Mosquito larval density was expressed as a percent of numbers of the species in the whole sample according to the formula, *D*°=°(*I*/*L*)°∗°100, where, *D* is density, *I* is the number of specimens of each mosquito species, and *L* is the total number of specimens [30]. The density classes were accepted following [30], satellite species (*D* < 1%); subdominant species (1 < *D* < 5%); dominant species (*D* > 5%).

All types of microbiota, that is, phytoplankton, bacterioplankton planktonic protists, and zooplankton occurrence frequencies were categorized as constant for species found in >50% of the habitats; common when found between 25% and 50% of the habitats; and accidental or rare species when found in <25% of the habitats [31].

The phytoplankton species richness of each sampling site (*α* diversity)—the number of species collected throughout the entire study period—was determined to calculate *α* medium, the average between *α* diversity for the sampling area of the same type of habitats. Gamma (*γ*) diversity was estimated using the total number of species from all samples and *β* diversity by measuring the species turnover using the *β* − 1 index [32] that measures the amount of the regional diversity that exceeds the mean alpha diversity and is calculated by the formula *β* − 1 = [(*S*/*α* mean) − 1]/[*N* − 1] × 100, where *S* is the number of species per each sampling site (total species richness), *α* mean is the mean alpha diversity (mean number of species) for each site in each period, and *N* is the number of sites of the period.

The phytoplankton species diversity was also estimated according to the indices of species richness (species per sample) (Shannon and Wiener [33]) and evenness [34].

## 4. Results

### 4.1. Species Composition of Mosquito Larvae and Microbiota

**Figure 2:**
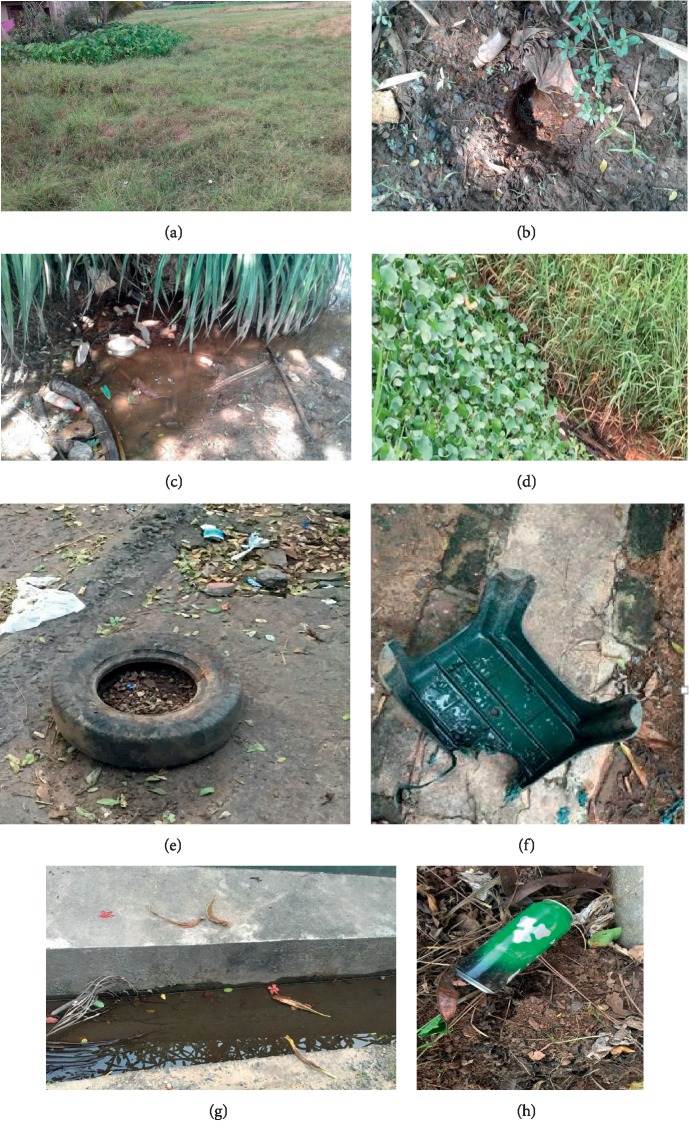
Common mosquito breeding habitats encountered ((a) rice field after harvest, (b) burrow pit, (c) stagnant water body, (d) marshy land, (e) discarded tire, (f)-plastic container, (g)-blocked drainage, (h)-metal container).

A total of 1495 mosquito larvae were collected, and nine species of mosquitoes belonging to four genera were identified from sixteen different types of mosquito breeding habitats (Table 2). Eight permanent macrotype mosquito breeding habitats, that is, rice fields, irrigation canals, blocked drainages, marshy lands, ponds, reservoirs, tank margins, and stagnant water bodies and eight temporary microtype mosquito breeding habitats, that is, tree holes, plastic containers, burrow pits, metal containers, discarded tyres, leaf litter, clay pots, and ornamentals were found across the study area (Table 2; Figures 2(a)–2(h)). A total of 4420 microbiota were recorded from all these habitats, and they represented 44 species belonging to ten phyla (Table 3). Nearly 30% of the habitats were represented by harvested rice fields. Four species of *Culex* mosquito larvae, namely, *Cx. quinquefasciatus*, *Cx. tritaeniorhynchus*, *Cx. Gelidus,* and *Cx.whitmorei* were detected in 56% of the breeding habitats mainly in rice fields and irrigation canals. *Aedes aegypti* and *Ae. albopictus* appeared in infrequent (20.1–40%) and moderate distribution (40.1–60%), respectively, in micro temporary habitats. *Anopheles subpictus*, *An. Vagus,* and *Mansonia uniformis* were sporadic in distribution (0–20%) and reported only from burrow pits, tank margins, and the rice fields, respectively. From the density values obtained, *Ae. albopictus*, *An. subpictus*, *Cx quinquefasciatus*, *Cx. tritaeniorhynchus*, *Cx. Gelidus,* and *Cx.whitmorei* were found to be the dominant species in their respective habitat types. The only species reported as subdominant in density was *Ae. aegypti*. Two satellite species, *An.vagus* and *Mansonia uniformis*, were found only in tank margins and rice fields, respectively.

### 4.2. Habitat Diversity and Occurrence of Microbiota Species

**Figure 3:**
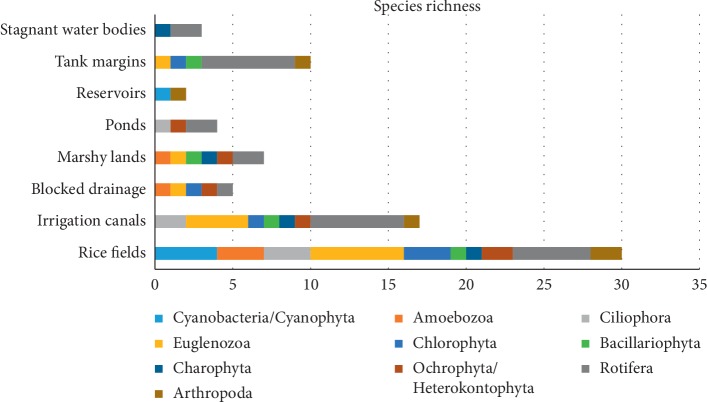
Number of species per macrotype permanent habitat during the study period (gamma diversity), distributed in taxonomic phyla.

**Figure 4:**
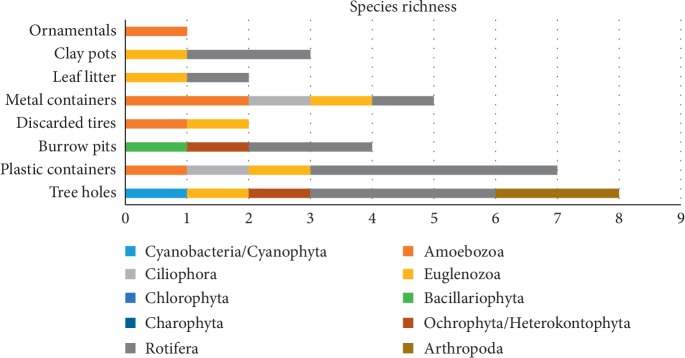
Number of species per microtype temporary habitat during the study period (gamma diversity), distributed in taxonomic phyla.

**Figure 5:**
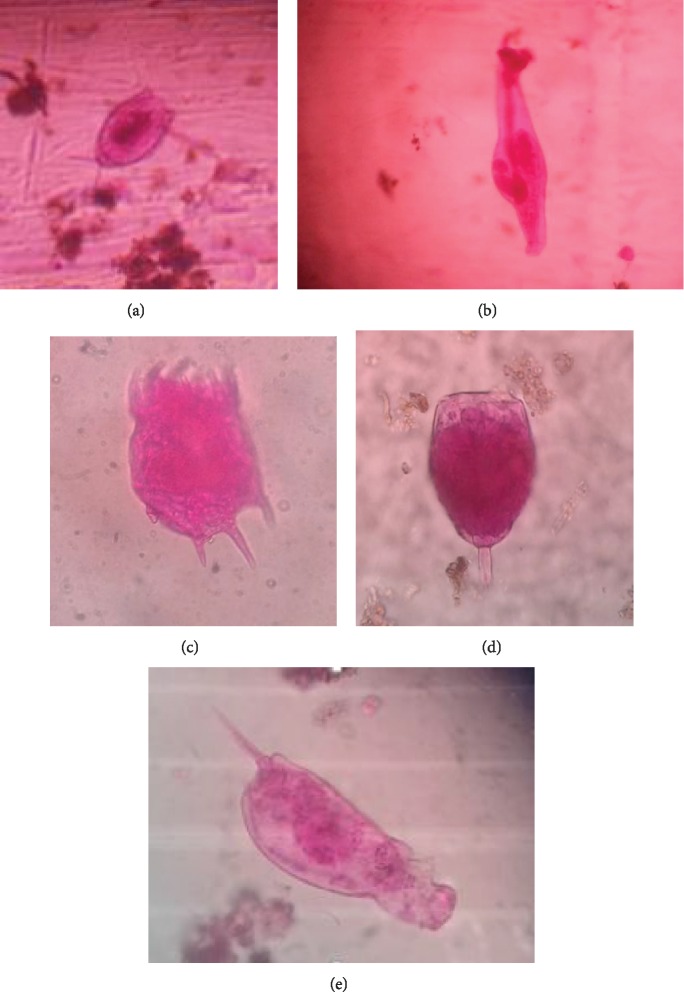
Microbiota species detected from mosquito breeding habitats, ×400 magnification ((a) *Lecane Luna*, (b) *Philodina citrina*, (c) *Keratella valga*, (d) *Monostyla hamata*, (e) *Diorella stylata*).

None of the microbiota taxa had constant frequency occurrence (FR% > 51%) in the mosquito breeding habitats during the study period. Frequency occurrence of twelve species/taxa, namely, *Arcella arenaria*, *Euglena acus*, *Euglena caudata*, *Gomphonema angustatum*, *Closterium* spp., *Pinnularia braunii*, *Lecane luna*, *Monostyla bulla*, *Philodina citrina*, *Notholca acuminata*, *Diorella stylata*, and *Canthocamptus staphylinus*, were in between 25% and 50%, hence considered as common species/taxa. The rest of the 32 species/taxa detected were uncommon/rare (frequency of occurrence < 24%; Table 3). It is important to note that 40% of the twelve microbiota species/taxa with common occurrence were specimens of rotifers. Rotifer species richness was highest in macro permanent habitats (Figure 3), as well as in micro temporary habitats (Figure 4). We observed a wide range of morphological variations among rotifers in this study (Figures 5(a)–5(e)). Among them, *Philodina citrina* and *Diorella stylata* comprised 23.5% and 22.5%, respectively, of the total rotifer population. The lowest species richness was recorded from three phyla, namely, Amoebozoa, Ciliophora, and Arthropoda, each represented only by three species, namely, *Arcella arenaria*, *Difflugia corona*, and *Acanthocystis aculeata*; *Paramecium bursaria*, *Vorticella microstoma*, and *Zoothamnium* spp.; and *Canthocamptus staphylinus*, *Daphnia magna*, and *Parastenocaris brevipes,* respectively.

Phytoplankton species distribution (gamma diversity value) across the habitat types during the study period for rice field habitats was 28 followed by irrigation canals and tank margins, *that is,* 17 and 10, respectively (Table 4; Table 3). Autotrophic species such as *Spirulina major*, *Phacus pleuronectes*, *P. curvicauda*, and species of the Phylum Chlorophyta had higher densities in rice field habitats and irrigation canals.

The highest microbiota diversity was observed in the rice field habitat. *Vorticella microstoma* and *Zoothamnium* sp. were among the highest abundant microbiota in these habitats (Table 3). In these habitats the mosquito larvae of *Cx. tritaeniorhynchus* was the most common species. The density of *Daphnia magna* which is a common freshwater cladoceran was very low in rice field habitats and reservoirs. *Arcella arenaria* and *Philodina citrina* were found in association with plastic and metal containers, ornaments, and clay pots which are ideal breeding grounds for the mosquito species, *Ae. aegypti* and *Ae. albopictus*.

### 4.3. Effect of Microbiota on Growth and Survival of Mosquito Larvae

Observations revealed that survival of *Cx. tritaeniorhynchus* mosquito larvae collected from rice field habitats was significantly reduced over the rearing period in the laboratory. None of the larvae pupated but they became moribund and died. Microscopic observations revealed the attachment of *Vorticella microstoma* (Ciliophora) in higher densities on the dead and moribund larvae and their multiplication on the host body. Individuals of *Zoothamnium* attached to mosquito larvae were also observed but there was not any detrimental effect on the survival of the host larvae. Autotrophic species such as *Spirulina major*, *Phacus pleuronectes*, *P. curvicauda*, and species of the Phylum Chlorophyta had higher densities in rice field habitats and irrigation canals. However, none of these species showed any direct effect on mosquito larvae development. Several species/taxa were observed inside the buccal cavity of 4^th^ instar larvae collected from the same habitat. Further, *Euglena geniculata*, *Closterium* spp., and *Pinnularia* spp. were served as diet organisms for *Aedes albopictus*, Cx *tritaeniorhynchu*s, and *Culex* spp. Larvae, respectively, collected from the same habitats.

## 5. Discussion

It was reported that rice fields in Kurunegala district harbor constant breeding places for Japanese encephalitis vector, *Cx. tritaeniorhynchus* [9, 35]. They regularly receive water through nearby irrigation canals for cultivation purpose. In this study, water samples collected from both types of habitats, that is, rice fields and irrigation canals, resulted in high densities of *Vorticella microstoma* causing a lethal effect on *Cx. tritaeniorhynchus*. Similarly, *Zoothamniun* species was reported from the same habitats in high densities, but no detrimental effect on mosquito larvae was observed. A previous study conducted in a different geographic area in Sri Lanka has reported that *Zoothamnium* sp. was the most prevalent microbiota in marshy lands resulting in a weak negative effect on *Cx. tritaeniorhynchus* larvae [19]. Also, Laird [36] has reported a dense attachment of *Zoothamnium* spp. that has caused the death of moribund individuals of mosquito larvae. However, reduction of larval survival was not observed due to attachment of *Zoothamnium* spp. in this study. Several other ciliate species, namely, *Lambornella stegomyia*, a naturally occurring ciliate found in earthenware pots occupied by *Stegomyia scutellaris* mosquito larvae [37] and *Chilodonella uncinata*, an endoparasitic ciliate of culicine and anopheline larvae found in rice fields, irrigation canals, marshy areas, ponds and pools [16], were not detected during this study. *Arcella arenaria*, *Difflugia corona*, *Acanthocystis aculeata,* and *Paramecium bursaria* were among heterotrophic protists present in a range of permanent macrohabitats including rice fields, irrigation canals, marshy lands, ponds, reservoirs, and tank margins and in two temporary microhabitats, that is, plastic/metal containers and clay pots in this study. Addicott [38] and Blaustein and Chase [17] reported that heterotrophic microeukaryotes such as protists and rotifers also are important components of nutritional resource for larvae, particularly in container habitats. Eleven species of rotifers that include *Philodina citrina*, *Lecane Luna*, *Monostyla bulla,* and *Notholca acuminata* were detected in a range of breeding habitats from rice fields, irrigation canals, marshy lands, ponds, reservoirs, and tank margins to container-type breeding habitats in the present study. Duguma, et al. [39] reported that increased abundance and diversity of microeukaryotes in the larval habitat significantly reduced the abundance of adult *Culex* mosquitoes owing to the competition for small size class aquatic microbial biomass.

*Canthocamptus staphylinus* and *Parastenocaris brevipes*, the two species of copepods, were detected in association with mosquito breeding habitats in this study. *Canthocamptus staphylinus* was a common species found in rice fields, irrigation canal, reservoirs, and tree holes whereas *Parastenocaris brevipes* was a rare species detected only from tree holes. The large size species of cyclopoid copepods are better effective biocontrol agents of mosquito larvae than that of other predatory invertebrates [14]. Several such species of copepods, namely, *Cyclops vernalis*, *Megacyclops formosanus*, *Mesocyclops aspericornis*, *M. edax*, *M. guangxiensis*, *M. longisetus,* and *M. thermocyclopoides*, were reported as active predators of young mosquito instars [40, 41]. Udayanga et al. [42] reported that *Mesocyclops leuckarti* had a successful predatory effect on *Ae. aegypti* and *Ae. albopictus* larvae, followed by *Mesocyclops crassus* collected from ponds, ditches, and other standing water sources of Gampaha and Kandy districts in Sri Lanka. Successful utilization of *Daphnia magna* to control *Culex pipiens* mosquito larvae in temporary water bodies was reported by Duquesne et al. [43]. *D. magna* was found rarely in this study.

An abundance of algal species usually provides favorable conditions for mosquito larval production. However, some algae have a lethal effect that can kill the mosquito larvae [15]. Krishnamurthy et al. [44] reported that some strains of *Microcystis aeruginosa* produce microcystin, a group of substances known to be toxic to various organisms. These authors also reported that *Microcystis* sp. has shown a significant negative effect on the development of mosquito larvae, where the larvae grown in the presence of alga were significantly smaller. *Microcystis* sp. was detected in lower densities from the rice fields and reservoirs associated with *Cx. quinquefasciatus* and *Cx. tritaeniorhynchus* mosquito species in this study. Howland [45] has reported that *Scenedesmus quadricauda* shows no signs of digestion in the mosquito gut. However, in the present study, high densities of two species of the same genus, *S. bijuga* and *S. armetus,* were detected from rice fields and blocked drainages which harbored *Cx.gelidus* and *Cx. quinquefasciatus,* respectively, without any negative effect on the larvae development [46].

## 6. Conclusion

A total of 44 microbiota species belonging to ten phyla were identified from a variety of mosquito breeding habitats in the Kurunegala district. *Vorticella microstoma* and *Zoothamniun* were found attached to the larvae of *Cx. tritaeniorhynchus,* working as possible agents against mosquito larvae breeding. *Vorticella microstoma* caused a lethal effect on *Cx. tritaeniorhynchus* larvae. The autotrophic protist, *Euglena geniculate*, *Closterium* spp., and *Pinnularia* spp. served as the diet items to mosquito larvae. The majority of the microbiota identified had no observable effect on mosquito larvae breeding.

## Figures and Tables

**Table 1:** Details of the climatic data of the Kurunegala district.

Month	Mean minimum temperature (°C)	Mean maximum temperature (°C)	Mean rainfall (mm)
January	21.6	29.8	90
February	21.1	31.7	49
March	22.5	34.0	90
April	24.1	33.5	227
May	25.1	32.1	191
June	24.8	30.1	119
July	24.4	29.9	80
August	24.4	30.5	70
September	24.0	31.1	122
October	23.4	31.0	341
November	22.9	30.4	326
December	22.4	29.6	125

Source: Natural Resources Management Center, Department of Agriculture, Sri Lanka and Department of Meteorology, Sri Lanka.

**Table 2:** Positivity of vector mosquito larvae in breeding habitats.

Breeding habitat	No. of samples	*Ae. aegypti*	*Ae. albopictus*	*An. subpictus*	*An.vagus*	*Cx. quinquefasciatus*	*Cx. tritaeniorhynchus*	*Cx.gelidus*	*Cx.whitmorei*	*Ma. uniformis*
Rice fields	**12**	−	−	−	−	+	+	+	+	+
Marshy lands	**2**	−	−	−	−	−	+	+	+	−
Ponds	**1**	+	+	−	−	−	−	−	−	−
Reservoirs	**2**	−	−	−	−	+	−	−	−	−
Tank margins	**1**	−	−	−	+	−	−	−	−	−
Irrigation canals	**5**	−	−	−	−	−	+	+	+	−
Blocked drainages	**1**	−	−	−	−	+	−	−	−	−
Tree holes	**3**	−	+	−	−	−	−	−	−	−
Plastic containers	**3**	+	+	−	−	−	−	−	−	−
Burrow pits	**2**	−	−	+	−	−	−	−	−	−
Tyres	**1**	+	−	−	−	−	−	−	−	−
Leaf litter	**1**	+	−	−	−	−	−	−	−	−
Metal containers	**2**	+	+	−	−	−	−	−	−	−
Ornamentals	**1**	−	+	−	−	+	−	−	−	−
Clay pots	**2**	−	+	−	−	−	−	−	−	−
Stagnant water bodies	**1**	−	+	−	−	−	−	−	−	−
No of larvae		**42**	**183**	**110**	**08**	**236**	**394**	**411**	**101**	**10**
Distribution (C)		31.25	43.75	6.25	6.25	25.0	18.75	18.75	18.75	6.25
Density (D)		2.86	12.31	7.4	0.53	15.88	26.51	27.65	6.79	0.67

(−) mosquito larvae not detected; (+) mosquito larvae positive habitats. C: distribution classes; C1: sporadic appearance (constancy 0–20%); C2: infrequent (20.1–40%); C3: moderate (40.1–60%); C4: frequent (60.1–80%); C5: constant (80.1–100%). *D*: density classes; satellite species (*D* < 1%); subdominant species (1 < *D* < 5%); dominant species (*D* > 5%).

**Table 3:** Occurrence of microbiota species in varying types of mosquito breeding habitats.

Taxonomic group of microbiota species/taxa	Breeding habitats															
	Rice fields	Irrigation canals	Blocked drainage canals	Tree holes	Marshy lands	Plastic containers	Ponds	Burrow pits	Discarded tyres	Metal containers	Reservoirs	Tank margins	Leaf litter	Clay pots	Ornamentals	Stagnant waterbodies
**Phy. Cyanobacteria/Cyanophyta**																
**F. Oscillatoriaceae**	2(1)	—	—	—	—	—	—	—	—	—	—	—	—	—	—	—
*Oscillatoria* spp.																
**F. Nostocaceae**	2(1)	—	—	2(1)	—	—	—	—	—	—	—	—	—	—	—	—
*Anabaena affinis*																
**F. Spirulinaceae**	120(2)	—	—	—	—	—	—	—	—	—	—	—	—	—	—	—
*Spirulina major*																
**F. Microcystaceae**	2(1)	—	—	—	—	—	—	—	—	—	2(1)	—	—	—	—	—
*Microcystis* spp.																
**Phy. Amoebozoa**																
**F. Arcellidae**	3(2)	—	—	—	—	12(1)	—	—	6(1)	6(1)	—	—	—	—	5(1)	—
*Arcella arenaria*																
**F. Difflugiidae**	—	—	2(1)	—	—	—	—	—	—	—	—	—	—	—	—	—
*Difflugia corona*																
*Acanthocystis aculeata*	10(1)	—	—	—	5(1)	—	—	—	—	28(1)	—	—	—	—	—	—
**Phy. Ciliophora**																
**F. Parameciidae**	—	—	—	—	—	8(1)	44(1)	—	—	12(1)	—	—	—	—	—	—
*Paramecium bursaria*																
**F. Vorticellidae**	860(5)	70(1)	—	—	—	—	—	—	—	—	50(1)	—	—	—	—	—
*Vorticella microstoma*																
**F. Zoothamniidae**	165(2)	30(1)	—	—	—	—	—	—	—	—	—					
*Zoothamnium* sp.																
**Phy. Euglenozoa**																
**F. Euglenaceae**	21(2)	—	—	—	—	—	—	—	1(1)	—	—	—	—	—	—	—
*Euglena variabilis*																
*Euglena acus*	—	—	—	—	—	6(2)	—	—	—	4(1)	—	—	2(1)	5(1)	—	—
*Euglena geniculata*	20(1)	—	—	15(1)	—	—	—	—	—	—	—	—	—	—	—	—
*Euglena gracilis*	45(2)	21(1)	—	—	—	—	—	—	—	—	—	—	—	—	—	—
*Euglena caudate*	35(2)	52(2)	26(1)	—	90(3)	—	—	—	—	—	—	10(1)	—	—	—	—
*Phacus pleuronectes*	116(1)	126(1)	—	—	—	—	—	—	—	—	—	—	—	—	—	—
*Phacus curvicauda*	176(3)	128(2)	—	—	—	—	—	—	—	—	—	—	—	—	—	—
**Phy. Chlorophyta**																
**F. Scenedesmaceae**	155(2)	—	—	—	—	—	—	—	—	—	—	55(1)	—	—	—	—
*Scenedesmus armetus*																
*Scenedesmus bijuga*	—	—	125 (1)	—	—	—	—	—	—	—	—	—	—	—	—	—
**F. Trebouxiophyceae ** *Crucigenia quadrata*	140(1)	—	—	—	—	—	—	—	—	—	—	—	—	—	—	—
**F. Volvocaceae**	250(1)	75(1)	—	—	—	—	—	—	—	—	—	—	—	—	—	—
*Pandorina morum*																
*Phy. Bacillariophyta*																
**F. Gomphonemataceae ** *Gomphonema angustatum*	84(6)	16(1)	—	—	5(1)	—	—	26(1)	—	—	—	25(1)	—	—	—	—
*Phy. Charophyta*																
**F. Closteriaceae**	25(2)	12(1)	—	—	5(1)	—	—	—	—	—	—	—	—	—	—	40(1)
*Closterium* spp.																
**Phy. Ochrophyta/Heterokontophyta**																
**Pinnulariaceae**	85(4)	35(2)	12(1)	2(1)	12(1)	—	—	10(1)	—	—	—	—	—	—	—	—
*Pinnularia braunii*																
*Pinnularia subsoralis*	10(1)	—	—	—	—	—	—	—	—	—	—	—	—	—	—	—
**Gloeobotrydaceae ** *Gloeobotrys limneticus*	—	—	—	—	—	—	20(1)	—	—	—	—	—	—	—	—	—
**Phy. Rotifera**																
**F. Lecanidae**	15(1)	12(1)	—	—	—	2(1)	—	—	—	—	—	2(1)	—	—	—	—
*Lecane Luna*																
*Monostyla bulla*	10(2)	24(2)	—	—	2(1)	—	—	—	—	—	—	2(1)	—	—	—	—
*Lecane unquitata*	—	—	1(1)	—	—	—	—	—	—	—	—	—	—	—	—	—
*Lecane lunaris*	—	—	—	—	—	—	12(1)	—	—	—	—	—	—	—	—	—
**F. Philodinidae**	14(2)	31(2)	—	—	—	6(2)	—	—	—	5(1)	—	30(1)	—	2(1)	—	—
*Philodina citrine*																
**F. Brachionidae**	22(1)	18(1)	—	—	—	—	—	—	—	—	—	—	—	—	—	—
*Keratella valga*																
*Notholca acuminata*	—	—	—	2(1)	—	6(1)	—	10(1)	—	—	—	—	2(1)	—	—	—
*Brachionus calyciflorus*	—	18(1)	—	—	—	—	—	—	—	—	—	—	—	—	—	—
*Brachionus falcatus*	—	—	—	—	—	—	—	—	—	—	—	—	—	—	—	4(1)
*Brachionus urceus*	—	—	—	10(2)	—	—	—	—	—	—	—	8(1)	—	—	—	—
*Euchlanis dilatata*	—	—	—	—	—	—	—	—	—	—	—	—	—	2(1)	—	—
**F. Lepadellidae**	—	—	—	—	—	3(1)	—	—	—	—	—	—	—	—	—	—
*Lepadella ovalis*																
**F. Asplanchnidae**	—	—	—	12(1)	—	—	—	—	—	—	—	15(1)	—	—	—	—
*Asplachna brightwellii*																
*Asplanchna priodonta*	—	—	—	—	—	—	—	5(1)	—	—	—	—	—	—	—	—
**F. Trichocercidae**	27(4)	29(3)	—	—	13(2)	—	1(1)	—	—	—	—	5(1)	—	—	—	10(1)
*Diorella stylata*																
Phy. Arthropoda																
**F. Canthocamptidae ** *Canthocamptus staphylinus*	24(3)	32(2)	—	6(1)	—	—	—	—	—	—	—	2(1)	—	—	—	—
**F. Daphniidae**	7(1)	—	—	—	—	—	—	—	—	—	2(1)	—	—	—	—	—
*Daphnia magna*																
**F. Parastenocarididae ** *Parastenocaris brevipes*	—	—	—	2(1)	—	—	—	—	—	—	—	—	—	—	—	—

(—) absence of microbiota; positive samples are given in parenthesis.

**Table 4:** Evenness (%), Shannon diversity, alpha (*α*), alpha medium, and beta (*β*) and gamma (*γ*) diversities of type of habitats.

Type of habitat	Sampling sites	*α* medium	*β*	*γ*	Shannon diversity	% evenness
Rice fields	1–12	5	42	28	123.93	35.17
Irrigation canals	13–17	4	81	17	52.88	17.56
Blocked drainages	18	6	0	5	14.18	74.53
Tree holes	19–20	3	83	8	19.39	29.41
Marshy lands	21	4	38	7	19.11	68.18
Plastic containers	22–26	4	38	7	14.6	27.9
Ponds	27	4	0	4	8.11	57.14
Burrow pits	28–30	3	34	4	6.25	50.98
Metal containers	31–32	3	67	5	9.43	50.91
Reservoirs	33–36	2	50	3	6.69	92.59
Tyres	37	2	0	2	2.1	85.71
Tank margins	38	10	0	10	28.96	35.71
Leaf litter	39–40	2	0	2	1.39	50
Clay pots	41	3	0	3	3.4	55.56
Ornamentals	42–43	2	0	1	0	100
Stagnant water bodies	44	3	0	3	4.59	74.07

## Data Availability

The datasets supporting the conclusions of this article are included within the article. Data will not be shared in any of the sources.
